# Influence of immune checkpoint inhibitor target and timing on combination treatment with antibody-based β-emitting targeted radionuclide therapy across tumor immunogenicity profiles

**DOI:** 10.3389/fimmu.2026.1872928

**Published:** 2026-08-04

**Authors:** Simone C. Kleinendorst, Egbert Oosterwijk, Mark Konijnenberg, Milou Boswinkel, Manon Gloudemans, Sylvia T.M. Wenker, Stijn Muselaers, Peter Mulders, Lyndsey Maffa, Michael F. O’Hara, Michael P. Wheatcroft, Kwame Twumasi-Boateng, Sandra Heskamp

**Affiliations:** 1 Department of Medical Imaging, Nuclear Medicine, Radboud University Medical Center, Nijmegen, Netherlands; 2 Department of Urology, Radboud University Medical Center, Nijmegen, Netherlands; 3 Department of Radiology and Nuclear Medicine, Erasmus MC Cancer Institute, Erasmus University Medical Center, Rotterdam, Netherlands; 4 Telix Pharmaceuticals Limited, Melbourne, VIC, Australia

**Keywords:** combination treatment, immune checkpoint inhibition, syngeneic animal model, targeted radionuclide therapy, tumor immunogenicity

## Abstract

**Introduction:**

Targeted radionuclide therapy (TRT) and immune checkpoint inhibition (ICI) could be a highly effective combination treatment due to potential synergistic effects. In this study, we investigated two clinically relevant antibody-based radiopharmaceuticals labeled with lutetium-177: [^177^Lu]Lu-DOTA-hG250 targeting carbonic anhydrase IX (CAIX) and rosopatamab tetraxetan ([^177^Lu]Lu-DOTA-rosopatamab) targeting prostate-specific membrane antigen (PSMA). We aimed to assess whether ICI target and treatment timing influence the efficacy of TRT+ICI combinations across three syngeneic murine tumor models with distinct immunogenicity profiles. Understanding these factors is critical for optimizing combination strategies.

**Methods:**

C57BL6 hPSMA transgenic mice bearing subcutaneous MC38-hPSMA or B16F10-hPSMA tumors were injected with [^177^Lu]Lu-DOTA-rosopatamab. Balb/c mice bearing subcutaneous renca-hCAIX tumors were injected with [^177^Lu]Lu-DOTA-hG250. Tumor growth and overall survival were evaluated for different combination treatments of hPSMA-TRT or hCAIX-TRT with aPD-1, aCTLA-4, or dual-ICI (*n* = 10 per group). To evaluate the impact of timing on therapeutic outcomes, different concurrent and sequential treatment schedules were compared.

**Results:**

In the MC38-hPSMA model, a subtherapeutic [^177^Lu]Lu-DOTA-rosopatamab dose combined with aPD-1 resulted in robust anti-tumor activity. aCTLA-4 monotherapy was already curative, precluding detection of a combination effect under the conditions tested. In contrast, in the B16F10-hPSMA model, only minimal benefit was seen when combining the same radiopharmaceutical with dual-ICI. The renca-hCAIX model demonstrated differential outcomes, responding most strongly to a subtherapeutic dose of [^177^Lu]Lu-DOTA-hG250 combined with dual-ICI, while combining with aPD-1 provided moderate benefit and with aCTLA-4 had no therapeutic benefit. Lastly, exploring treatment timing in the MC38-hPSMA and renca-hCAIX models revealed only limited impact on therapeutic outcome under the assessed conditions.

**Discussion:**

These findings indicate that ^177^Lu-labeled antibodies can synergize with ICI at subtherapeutic activity levels in certain settings, supporting their potential to enhance efficacy while potentially reducing toxicity. Combination outcomes varied across tumor models and ICI targets and were likely influenced by both biological and experimental context. The impact of treatment timing appeared limited within the experimental conditions evaluated. These findings should be further explored in preclinical models incorporating mechanistic analyses, while the impact of treatment timing warrants additional investigation in clinically relevant settings. These insights highlight key factors for optimizing TRT+ICI strategies in future clinical studies.

## Graphical Abstract

**Figure:**
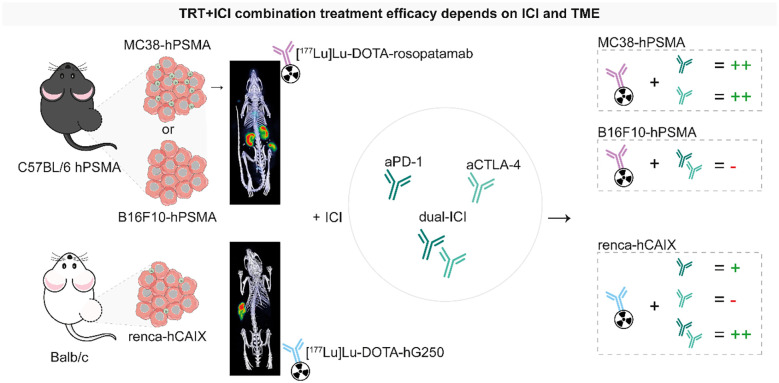

## Introduction

Targeted radionuclide therapy (TRT) is a systemic treatment using a tumor-targeting radiopharmaceutical to deliver therapeutic radiation to target-positive tumor lesions. The FDA approvals of [^177^Lu]Lu-DOTATATE and [^177^Lu]Lu-PSMA-617 have increased interest in leveraging TRT as a treatment modality in cancer (1, 2). However, durable clinical benefit is limited to subsets of patients, highlighting the need for strategies to enhance efficacy (3). A promising approach to improve TRT efficacy is combination therapy, with growing interest in leveraging the immunomodulatory effects of radiation to enhance anti-tumor immune responses (4, 5).

TRT has been shown to exert immunomodulatory effects, including activation of interferon signaling and remodeling of the tumor microenvironment (TME) (6, 7). Additionally, irradiation can induce *in situ* auto-vaccination via the release of danger signals and tumor-associated antigens (8). Given these effects, TRT represents a promising combination partner for immunotherapy, such as immune checkpoint inhibitor (ICI) treatment. ICIs block signals that inhibit T-cell activity (9). The resulting (re)activation of T cells may be enhanced by the immune-stimulating effects of TRT.

An increasing number of preclinical and clinical studies are investigating the therapeutic potential of TRT combined with ICI. Preclinical research has provided ample evidence for synergistic anti-tumor effects (10), and early-phase clinical trials exploring TRT+ICI combinations have reported promising results (11, 12). Since the effectiveness of such combination therapies may depend on key variables, including the target of ICI used and the timing of its administration relative to TRT, optimizing these parameters is critical to maximize therapeutic synergy and inform rational clinical trial design.

Therefore, this study aimed to investigate whether the ICI target (aPD-1, aCTLA-4, or their combination) and timing influence the efficacy of combined TRT+ICI therapy. To this end, we employed two clinically relevant radiopharmaceuticals, both consisting of monoclonal antibodies labeled with the beta-emitter lutetium-177 (^177^Lu): humanized girentuximab ([^177^Lu]Lu-DOTA-hG250) and rosopatamab tetraxetan ([^177^Lu]Lu-DOTA-rosopatamab). hG250 targets carbonic anhydrase IX (CAIX), an antigen overexpressed in the majority of clear cell renal cell carcinomas (ccRCC) (13). As an imaging PET agent, G250 radiolabeled with zirconium-89 (i.e., [^89^Zr]Zr-girentuximab) has demonstrated high accuracy for the detection and characterization of ccRCC (14), and its therapeutic potential with ^177^Lu has been demonstrated in clinical studies (15, 16). However, myelotoxicity at higher doses limited re-treatment and further clinical implementation, prompting preclinical evaluation of low-dose [^177^Lu]Lu-DOTA-hG250 in combination with ICI. Our previous work showed synergistic anti-tumor responses following [^177^Lu]Lu-DOTA-hG250+ICI treatment in an RCC model (6).

[^177^Lu]Lu-DOTA-rosopatamab targets prostate-specific membrane antigen (PSMA), a well-established TRT target in metastatic castration-resistant prostate cancer (mCRPC) (1, 17). The clinical relevance of PSMA-TRT has been further validated by the FDA-approved agent [^177^Lu]Lu-PSMA-617. Early-phase clinical studies with [^177^Lu]Lu-DOTA-rosopatamab have demonstrated a favorable safety profile and signs of efficacy, supporting its ongoing evaluation in a phase III trial (NCT04876651) (18, 19).

To investigate whether ICI target and timing influence treatment outcomes, we studied combinations of [^177^Lu]Lu-DOTA-hG250 or [^177^Lu]Lu-DOTA-rosopatamab with ICI in three syngeneic murine tumor models with distinct immunogenicity profiles. Balb/c mice bearing hCAIX-expressing renca tumors were used to study CAIX-TRT+ICI, representing a moderately immunogenic tumor (6, 20). C57BL/6-hPSMA transgenic mice bearing hPSMA-expressing MC38 or B16F10 tumors were used to study PSMA-TRT+ICI, representing highly and poorly immunogenic tumors, respectively (20, 21).

## Materials and methods

More detailed methods can be found in the **Supplementary Materials**.

### Antibodies and cell lines

hCAIX-directed humanized IgG1 monoclonal antibody girentuximab (hG250) and DOTA-conjugated hPSMA-directed humanized IgG1 monoclonal antibody DOTA-rosopatamab were provided by Telix Pharmaceuticals (Melbourne, Australia). Anti-murine PD-1 and anti-murine CTLA-4 were purchased from Bio X Cell, Lebanon, NH, USA [BE0146-clone RMP1-14, and BE0032-clone UC10-4F10-11 (hG250 studies) or BE0131-clone 9H10 (rosopatamab studies)]. Renca-hCAIX cells were used as previously described (6), MC38-hPSMA cells were licensed from Biocytogen, Beijing, China, and B16F10-hPSMA cells were custom-engineered by GenScript and provided by Telix Pharmaceuticals.

### Conjugation, radiolabeling, and quality control

hG250 was conjugated with DOTA, and hG250-DOTA and rosopatamab-DOTA were labeled with ^177^Lu as described in the **Supplementary Methods**. Labeling efficiency was determined by instant thin-layer chromatography (ITLC) or high-performance liquid chromatography (HPLC) and exceeded 90% for both antibodies. The immunoreactive fraction of both antibodies exceeded 70%.

### Animal models

All animal procedures were approved by the Radboudumc Animal Welfare Body (renca-hCAIX model: female BALB/cAnNRj or BALB/cJRj mice bearing renca-hCAIX tumors) or the Perceptive Discovery Institutional Animal Care and Use Committee (IACUC) (MC38-hPSMA and B16F10-hPSMA models: female C57BL/6-Folh1tm1(FOLH1)/Bcgen hPSMA transgenic mice bearing MC38-hPSMA or B16F10-hPSMA tumors). Body weight and tumor size were monitored three times (renca-hCAIX) or twice (MC38-hPSMA and B16F10-hPSMA) per week until the end of the study or until a humane endpoint was reached [tumor volume ≥ 1.5 cm^3^ (renca-hCAIX) or ≥ 2 cm^3^ or ≥ 20 mm in any direction (MC38-hPSMA/B16F10-hPSMA), tumor ulceration, >15% body weight loss within 2 days, >20% body weight loss compared with the start weight, or severe clinical deterioration]. TRT-target expression was confirmed by immunohistochemistry for all tumor models (See (6) and **Supplementary Figure 1**).

Detailed descriptions of the tumor models, experimental procedures, applied humane endpoints, and animal characteristics are provided in the **Supplementary Methods** and **Supplementary Table 1**.

### Biodistribution studies

*In vivo* biodistribution of [^177^Lu]Lu-DOTA-rosopatamab was studied by injecting three MC38-hPSMA tumor-bearing mice with 13 MBq [^177^Lu]Lu-DOTA-rosopatamab and obtaining SPECT/CT scans on days 3 and 7 postinjection under general anesthesia. Mice were sacrificed on day 7; tumor and normal tissues were harvested and weighed; and radioactivity was measured in a γ-counter. *In vivo* biodistribution of [^177^Lu]Lu-DOTA-hG250 was studied by injecting three renca-hCAIX tumor-bearing mice with 20 MBq [^177^Lu]Lu-DOTA-hG250 and obtaining SPECT/CT scans on day 3 postinjection under general anesthesia. *Ex vivo* biodistribution was studied by injecting five mice per time point with 0.2 MBq [^177^Lu]Lu-DOTA-hG250. Mice were sacrificed on days 1, 3, and 7 postinjection to quantify uptake as described above.

### Animal therapy experiments

**Figure 1:**
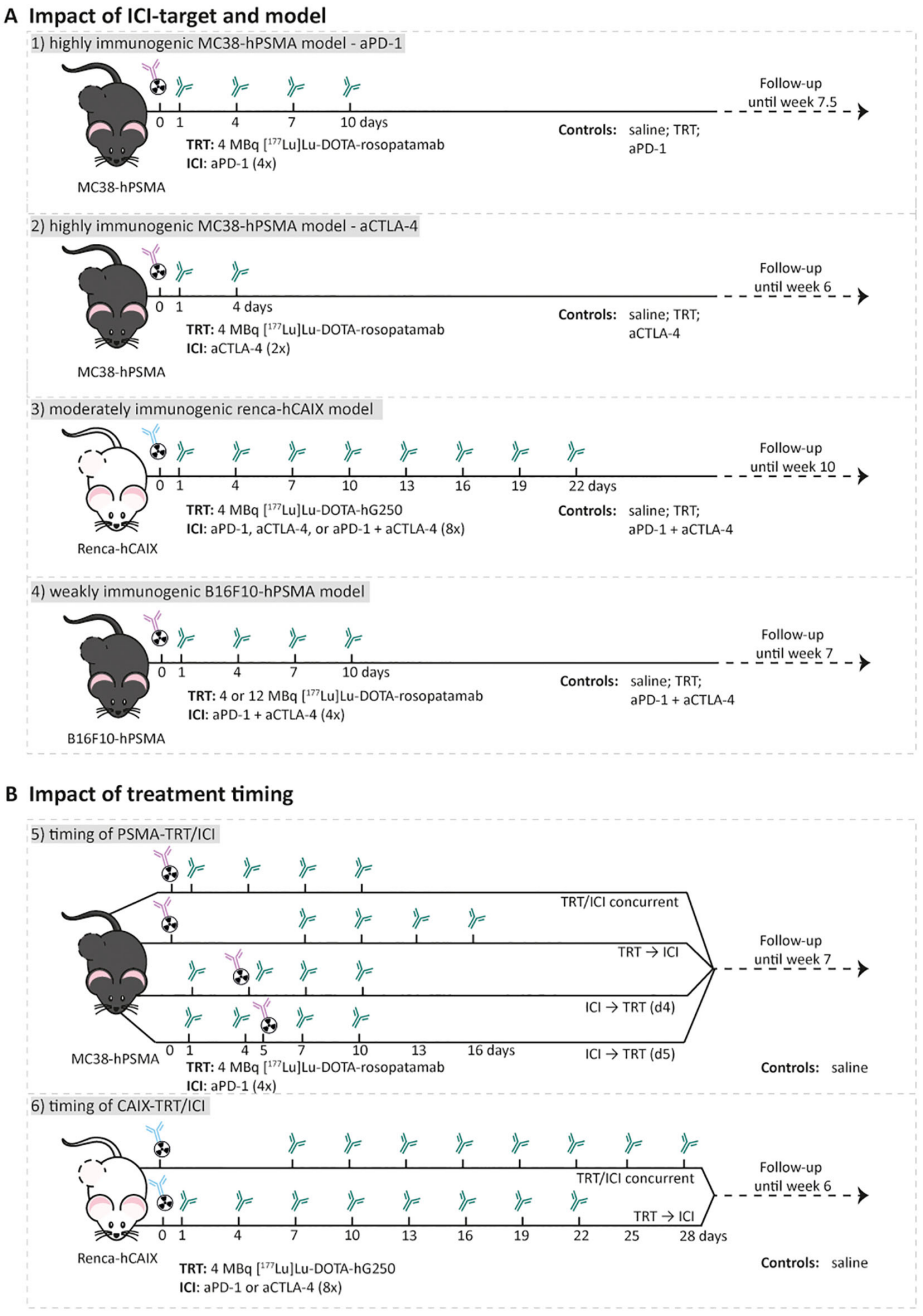
Overview of the therapeutic animal studies evaluating the effects of **(A)** ICI target and model and **(B)** treatment timing on the efficacy of ICI combined with hPSMA-TRT ([^177^Lu]Lu-DOTA-rosopatamab) in MC38-hPSMA or B16F10-hPSMA tumor-bearing mice, or hCAIX-TRT ([^177^Lu]Lu-DOTA-hG250) in Renca-hCAIX tumor-bearing mice.

Tumor-bearing mice were randomized into groups of 10 animals based on tumor volume to receive the treatments as follows: all TRT injections entailed intravenous injection of [^177^Lu]Lu-DOTA-hG250 (5 µg in 200-µL saline) or [^177^Lu]Lu-DOTA-rosopatamab (32 ± 2 µg in 150-µL saline) on day 0 unless indicated otherwise. All ICI injections entailed intraperitoneal injections of 200 µg aPD-1 and/or 200 µg aCTLA-4 in 200-µL saline every 3 days, starting from day 1 unless indicated otherwise. Control injections were done at the same volumes and administration routes and contained saline only. Experimental designs, including treatment groups and timing schedules, are shown in **Figure 1** and detailed in the **Supplementary Methods**.

### Statistical analyses

Tumor growth was quantified as normalized area under the curve (nAUC), which is the area under the tumor growth curve of single animals normalized to animal lifetime. Differences in nAUC between groups were compared using the Kruskal–Wallis test with Dunn’s multiple comparisons test. Complete response was defined as the absence of a palpable or caliper-measurable tumor at study endpoint. All survival data were visualized as a Kaplan–Meier plot. Restricted mean survival time (RMST) was calculated, and RMST ratios between the groups were compared using the ‘survRM2’ package in R (22). Other analyses were performed with Graphpad Prism, version 5.03. A p-value of <0.05 (tumor growth) or a Bonferroni-corrected α-value (survival) was considered statistically significant.

## Results

### Tumor uptake of [^177^Lu]Lu-DOTA-rosopatamab in MC38-hPSMA and [^177^Lu]Lu-DOTA-hG250 in renca-hCAIX

**Figure 2:**
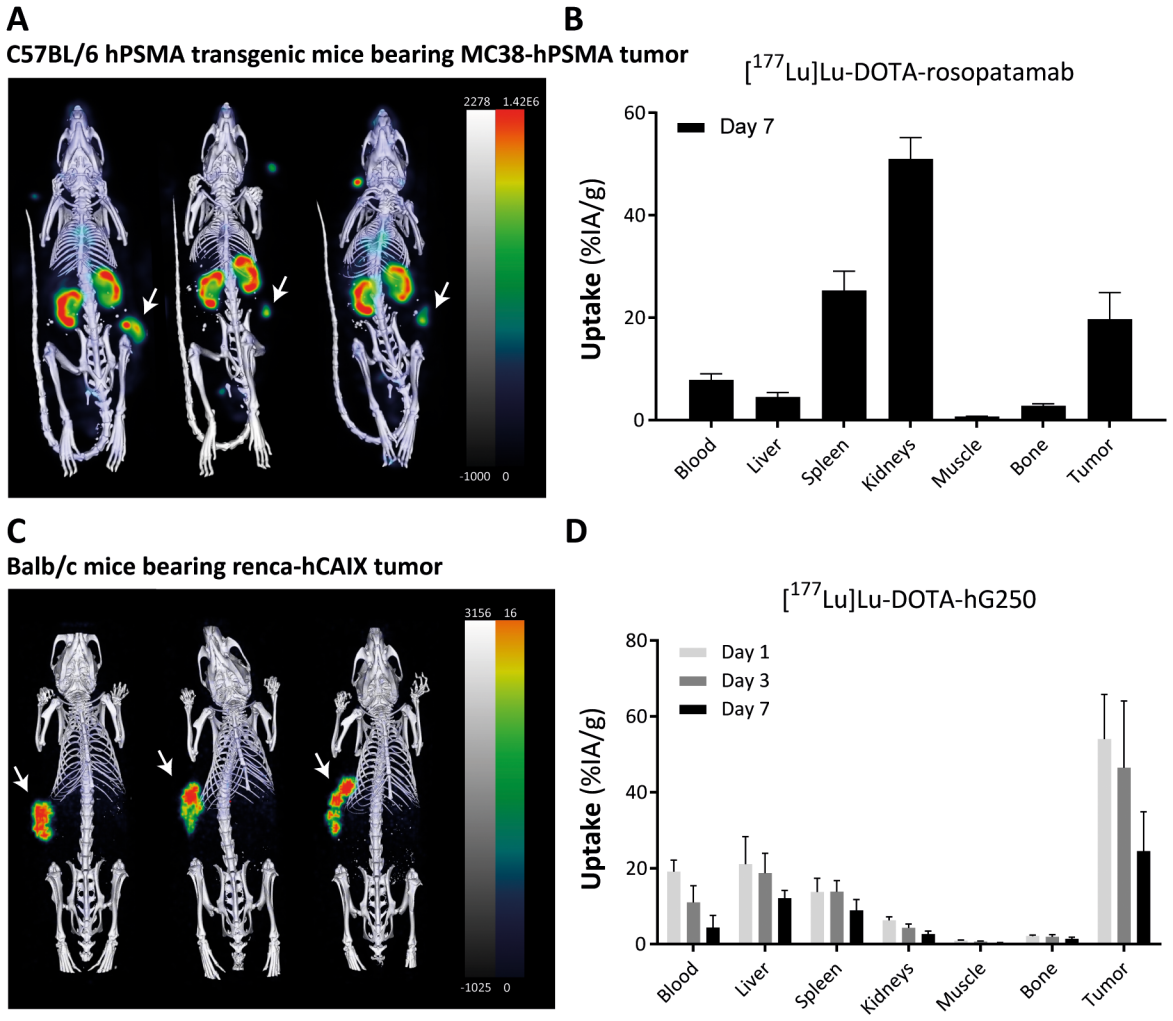
Biodistribution of **(A, B)** [^177^Lu]Lu-DOTA-rosopatamab in MC38-hPSMA tumor-bearing C57BL/6-hPSMA transgenic mice, and **(C, D)** [^177^Lu]Lu-DOTA-hG250 in renca-hCAIX tumor-bearing balb/c mice. **(A, C)** Representative SPECT-CT scans 3 days post-administration of 13 MBq [^177^Lu]Lu-DOTA-rosopatamab or 20 MBq [^177^Lu]Lu-DOTA-hG250. Arrows indicate tumors. **(B, D)** *Ex vivo* uptake values of 13 MBq [177Lu]Lu-DOTA-rosopatamab (group mean+SD, n=3) on day 7 postinjection, and of 0.2 MBq [^177^Lu]Lu-DOTA-hG250 (group mean+SD, n=5) on days 1, 3, or 7 postinjection.

The highest uptake of [^177^Lu]Lu-DOTA-rosopatamab was observed in the kidneys [51 ± 2.4% of injected activity per gram (%IA/g)], spleen (25.3 ± 2.2%IA/g), and MC38-hPSMA tumors (19.7 ± 3%IA/g) (**Figures 2A, B**; **Supplementary Table 2**). Other organs showed relatively lower uptake (liver: 4.53 ± 0.5%IA/g, and bone: 2.77 ± 0.24%IA/g).

The highest uptake of [^177^Lu]Lu-DOTA-hG250 was observed in renca-hCAIX tumors (day 1: 54.0 ± 11.7%IA/g, day 3: 46.5 ± 17.6%IA/g, and day 7: 24.5 ± 10.4%IA/g) (**Figures 2C, D**; **Supplementary Table 3**). Mean blood levels were the highest on day 1 (19.1 ± 3.0%IA/g) and gradually decreased over time. Most organs showed low uptake with the exception of the liver and spleen, both demonstrating tracer uptake at all time points (21.1 ± 7.2 and 13.9 ± 2.9%IA/g maximum liver and spleen uptake, respectively). Renca-hCAIX tumor-absorbed dose was estimated at 5.6 ± 4.1 Gy/MBq (**Supplementary Figure 2**).

### TRT+ICI combination therapy suggests model- and ICI-target-dependent differences in therapeutic efficacy

For all therapeutic efficacy experiments described below, quantitative outcome parameters and statistical results are summarized in **Supplementary Table 4**, and body weight data for all experiments are shown in **Supplementary Figures 3** and **S4**. In all experiments, no apparent signs of treatment-related toxicity were observed over the course of the experiments, based on longitudinal body weight measurements, daily assessment of animal wellbeing, and macroscopic assessment for any overt abnormalities at necropsy.

**Figure 3:**
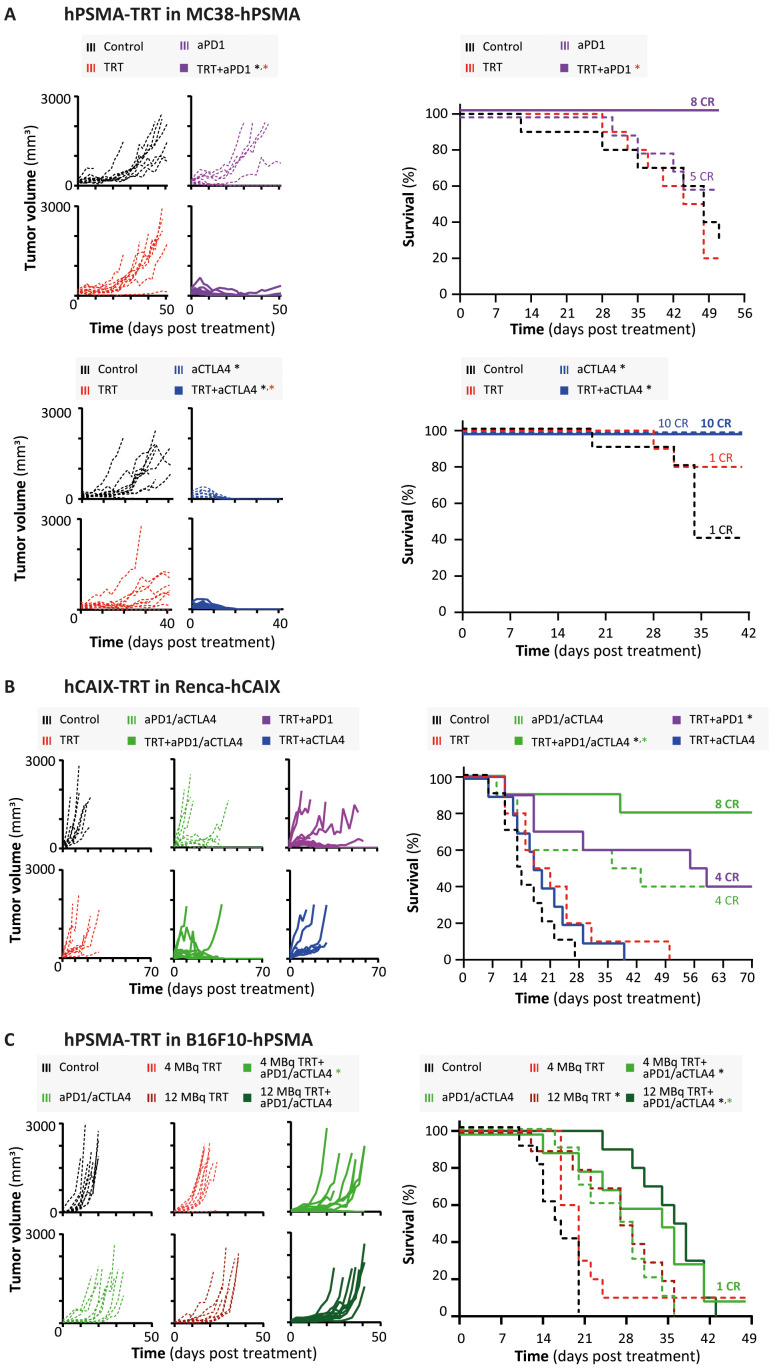
Therapeutic efficacy evaluated by tumor growth (left panels) and overall survival (right panels) of **(A)** 4 MBq [^177^Lu]Lu-DOTA-rosopatamab (hPSMA-TRT) with aPD-1 (4x) or aCTLA-4 (2x) in MC38-hPSMA tumor-bearing mice, **(B)** 4 MBq [^177^Lu]Lu-DOTA-hG250 (hCAIX-TRT) with aPD-1, aCTLA-4 or dual-ICI (8x) in renca-hCAIX tumor-bearing mice, and **(C)** 4 or 12 MBq [^177^Lu]Lu-DOTA-rosopatamab (hPSMA-TRT) with dual-ICI (4x) in B16F10-hPSMA tumor-bearing mice. Colored asterisks indicate statistically significant differences in nAUC (tumor growth) or Kaplan–Meier survival versus the group of the corresponding color. CR, complete response.

Combination treatment of hPSMA-TRT with aPD-1 was effective in the MC38-hPSMA tumor model, resulting in 8/10 complete responses (**Figure 3A**). At the time of data cutoff, combination therapy significantly delayed tumor growth, compared with control and TRT-treated (p<0.05), but not ICI-treated animals. Overall survival was also significantly prolonged after combination compared with TRT alone (p<0.01). TRT in combination with aCTLA-4 and aCTLA-4 monotherapy were indistinguishable, with both showing 10/10 complete response rates and significant anti-tumor efficacy/survival compared to control and TRT-treated animals (p<0.05/p<0.01) (**Figure 3B**).

Consistent with our previous findings (6), hCAIX-TRT combined with dual-ICI (defined as aPD-1+aCTLA-4 throughout the study) significantly inhibited tumor growth (p<0.05) and improved survival (p<0.007) compared with controls in the renca-hCAIX model, resulting in 8/10 complete responses (**Figure 3B**). Additionally, mice treated with TRT+dual-ICI had significantly better survival than TRT+aCTLA-4-treated mice (p<0.007), whereas the latter treatment did not provide a therapeutic benefit. In contrast, TRT+aPD-1 treatment demonstrated moderate anti-tumor efficacy (4/10 complete responders), with a significant difference in tumor growth compared with control animals (p<0.05). In a subsequent study, we compared TRT+aPD-1 and TRT+aCTLA-4 with their respective ICI monotherapies (**Supplementary Figure 5**). No significant differences in tumor growth or survival were observed between groups, although the TRT+aPD-1 group underperformed compared to earlier experiments, possibly due to lower overall animal wellbeing (**Supplementary Figures 5C, S6**). Nevertheless, complete tumor responses occurred only in the combination groups, again suggesting the benefit of combining ICI with TRT.

Thus far, we observed that ^177^Lu-based TRT combined with aPD-1 resulted in substantial responses in the MC38-hPSMA model, but less so in the renca-hCAIX model, where dual-ICI was required for optimal responses. To further evaluate these patterns, we selected the third model, B16F10-hPSMA, characterized by low baseline immunogenicity and radioresistance. Given the expected reduced treatment sensitivity compared with the other models, we selected dual-ICI and two activity levels, namely, 4 and 12 MBq hPSMA-TRT. The B16F10-hPSMA model demonstrated only limited therapeutic efficacy of combined hPSMA-TRT+dual-ICI (**Figure 3C**). Combination treatment with 4 MBq TRT+ICI significantly reduced tumor growth compared with TRT monotherapy (p<0.05), and dual-ICI + 4 MBq or 12 MBq combination treatment improved survival compared with untreated animals (p<0.0056). Combination treatment with 12 MBq TRT+ICI also outperformed ICI monotherapy. However, the overall benefit of combination therapy was markedly less pronounced than in the other models. Only 1/10 complete responses were achieved with 4 MBq combination therapy, while 12 MBq combination did not induce any complete responses.

### Impact of the timing of TRT and ICI on therapeutic efficacy

**Figure 4:**
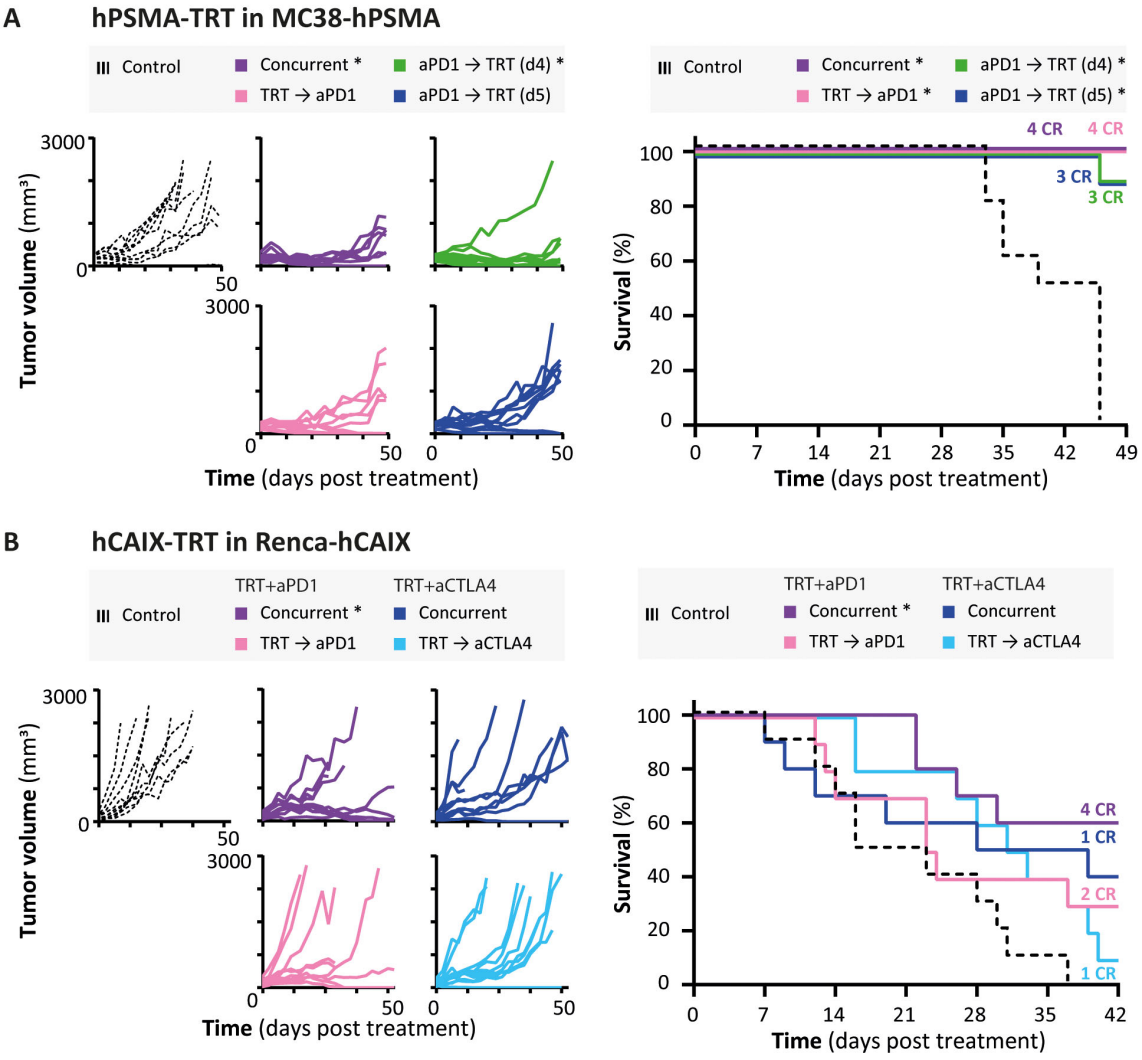
Therapeutic efficacy evaluated by tumor growth (left panels) and overall survival (right panels) of **(A)** 4 MBq [^177^Lu]Lu-DOTA-rosopatamab (hPSMA-TRT) with aPD-1 (4x) at concurrent or TRT → ICI timing, or TRT given during aPD-1 cycles at day 4 or 5 in MC38-hPSMA tumor-bearing mice and **(B)** 4 MBq [^177^Lu]Lu-DOTA-hG250 (hCAIX-TRT) with aPD-1 or aCTLA-4 (8x) starting at 1 day post-TRT (concurrent) or 7 days post-TRT (TRT → ICI) in renca-hCAIX tumor-bearing mice. Colored asterisks indicate statistically significant differences of nAUC (tumor growth) or Kaplan–Meier survival versus the group of the corresponding color. CR, complete response.

To assess whether treatment timing affects the therapeutic efficacy of TRT+ICI, we compared concurrent administration as applied in the experiments described above (ICI initiation 1 day post-TRT) with sequential schedules: ICI initiation 7 days post-TRT (TRT → ICI), and TRT on day 4 or 5 after ICI initiation (ICI → TRT (d4/5) in the MC38-hPSMA model (**Figure 4A**). All combination treatments significantly improved survival compared with untreated animals (p<0.0056), and tumor growth was significantly reduced after concurrent timing and when TRT was given 4 days after the first ICI dose (p<0.05).

Similarly, the efficacy of concurrent and TRT → ICI administration schedules of hCAIX-TRT with aPD-1 or aCTLA-4 were compared in renca-hCAIX tumor-bearing mice (**Figure 4B**). The results for concurrent TRT+aPD-1 and TRT+aCTLA-4 treatment groups were consistent with those described in **Figure 3B**, with only TRT+aPD-1 significantly reducing tumor growth (p<0.05) and improving survival (p<0.0083) compared with untreated animals. Notably, there were no significant differences in tumor growth or survival between the different schedules of administration.

## Discussion

This study evaluated whether ICI target and treatment timing influence the therapeutic efficacy of combined TRT and ICI, using two clinically relevant radiopharmaceuticals across three syngeneic murine tumor models with distinct immunogenicity profiles.

Our results provide compelling proof-of-concept that a subtherapeutic activity of hCAIX- or hPSMA-TRT combined with ICI exhibits promising anti-tumor efficacy. This was demonstrated across two mouse strains of varying genetic backgrounds, in three immunogenically distinct tumor models, and using different ICI schedules. The synergistic potential of TRT and ICI is increasingly recognized, as reflected by several clinical trials, but conflicting outcomes have been reported. For example, an early clinical study combining [^177^Lu]Lu-PSMA-617 with pembrolizumab has shown signs of immune activation and anti-tumor activity in mCRPC patients (11), even though prostate cancer is generally perceived as poorly immunogenic (23). However, clinical trials combining the α-emitter radium-223 dichloride (^223^Ra) with ICI have not demonstrated clear clinical benefit for mCRPC (24, 25), although comprehensive immunophenotyping of circulating immune cells reported substantial immunomodulatory effects of ^223^Ra monotherapy (26). These findings underscore the need to evaluate various parameters of TRT+ICI therapy.

Our findings highlight the importance of TRT dosing strategy. We selected a TRT activity level which in itself showed no or only limited anti-tumor efficacy as monotherapy, but could potentiate ICI efficacy in two of three models tested. Biodistribution analyses confirmed effective tumor targeting in MC38-hPSMA and renca-hCAIX tumors. For B16F10-hPSMA, biodistribution data are not available, but based on the pronounced PSMA expression observed by IHC, PSMA-mediated tumor uptake would be expected (**Supplementary Figure 1**). Despite this, combined hPSMA-TRT and dual-ICI failed to induce substantial therapeutic benefit in this model. However, in the absence of biodistribution and dosimetry data for this model, we cannot distinguish whether the limited therapeutic efficacy arises from biological resistance associated with a poorly immunogenic TME or from insufficient radiopharmaceutical uptake and tumor-absorbed dose.

The type of cell death upon radiation (which is likely dose-dependent) influences immunological outcome (27, 28). Therefore, in addition to the preclinical work performed so far, clinical studies should evaluate lower activities in combination with ICI, because the optimal activity level resulting in tumor ablation may not be equal to the activity level needed for immune modulation. Furthermore, incorporating dosimetric analysis will be essential to define the optimal dose range for immunogenic effects and to determine how low the tumor-absorbed dose can be while maintaining synergy. This dose reduction is particularly desirable in view of possible reductions in toxicities such as renal and hematological toxicities from TRT. In this context, it should be noted that pronounced renal uptake of [^177^Lu]Lu-DOTA-rosopatamab was observed in MC38-hPSMA tumor-bearing mice, which is potentially attributable to physiological PSMA expression in the kidneys (29). However, emerging clinical data for [^177^Lu]Lu-DOTA-rosopatamab indicate that kidney-absorbed doses remain within acceptable limits and are generally not associated with significant nephrotoxicity, supporting the translational feasibility of this approach. Similarly, given the clinical burden of immune-related adverse events, determining whether reduced ICI dosing and frequency are feasible in combination with TRT remains an open and clinically important question. Importantly, to substantiate the potential benefit of such dose-reduction strategies, future studies should incorporate comprehensive and longitudinal toxicity assessments, including hematological and organ-specific analyses, to more fully characterize the safety profile of these combination regimens.

Beyond this proof-of-concept, our results suggest model- and ICI-target-dependent outcomes. In MC38-hPSMA, hPSMA-TRT combined with PD-1 blockade was curative. We were unable to assess the efficacy of hPSMA-TRT/aCTLA-4 combination therapy under the tested dosing conditions, as aCTLA-4 monotherapy was already curative. In renca-hCAIX, responses differed by ICI regimen, with hCAIX-TRT combined with aPD-1 showing moderate efficacy, with aCTLA-4 no meaningful benefit, and with dual-ICI the most pronounced effect. These differences likely reflect a convergence of tumor-intrinsic features, host immune background, and immune checkpoint pathway biology. Apart from the tumor-related immune microenvironment, differences in mouse strain (C57BL/6 for MC38-/B16F10-hPSMA and Balb/c for renca-hCAIX) likely contribute to TRT+ICI response due to their differences in Th1- and Th2 immune response (30). Elucidating these determinants is particularly important given reports that combined TRT+ICI using beta-emitters tends to perform best in settings with pre-existing adaptive immune activity, whereas α-emitters can promote immune priming and may be advantageous in immune-cold tumors (31, 32).

Furthermore, apart from the distinct functions of the PD-1/PD-L1 and CTLA-4 pathways, cross-model comparisons of the effect of aCTLA-4 need to be approached with caution, given the different aCTLA-4 clones used. The 9H10 clone used in hPSMA-models has been associated with Fc-dependent depletion of regulatory T cells and is generally considered more potent. It was intentionally selected given the expected immune-refractory nature of the B16F10 model. In contrast, the UC10-4F10–11 clone used in renca-hCAIX primarily acts through checkpoint blockade without substantial depletion (33). Accordingly, CTLA-4 differences in anti-tumor efficacy observed between models may partly reflect ICI clone-specific mechanisms rather than inherent differences in tumor biology or treatment combinations. Despite this, the observation that TRT+dual-ICI in the renca-hCAIX model achieved more pronounced therapeutic effects than those observed in the B16F10-hPSMA model, despite the use of a less potent CTLA-4 clone, suggests that tumor immunogenicity may be an important determinant of response.

Consistent with our observations, other studies have also reported variable outcomes depending on the ICI used with TRT (34–39), and a recent systematic review suggested that TRT+aPD-1 blockade may be less effective than TRT+aPD-L1 blockade (10). To disentangle the relative contributions of biological determinants (e.g., tumor- and host-background and checkpoint pathway), future research should test the same TRT while systematically varying ICIs across well-characterized models. Such efforts would further benefit from harmonized experimental designs to enable direct comparisons, a consideration that was not uniformly achievable in the present exploratory study. Additional approaches such as varying implantation sites of the same tumor line to induce divergent TMEs (40), together with mechanistic cell, spatial, and genomic profiling, may further clarify the underlying biology.

Lastly, our assessment of treatment timing suggested only minimal impact on therapeutic efficacy under the conditions evaluated. However, while preclinical studies often schedule ICI dosing in close succession to TRT, this approach may not fully capture clinically relevant dynamics. The value of rapidly growing subcutaneous models, such as those used here and in the literature (37, 38, 41–44), for evaluating sequencing strategies may be limited, because any (especially subtle) effects of treatment timing could be masked by aggressive tumor kinetics. The dynamics of tumor growth and immune responses in mice can differ substantially from human disease. Nevertheless, preclinical systems remain valuable for mechanistic insights, for example, for profiling dynamic biological TME changes following TRT or ICI. Models with slower tumor growth, such as spontaneous or genetically engineered mouse models, may provide a better platform for assessing the interplay among TRT+ICI timing, tumor behavior, and immune cell activation (45). Ultimately, however, optimal sequencing must be defined in clinical trials. Notably, one clinical study combining [^177^Lu]Lu-PSMA-617 with pembrolizumab tested three schedules: TRT administered 28 days before, concomitant with, or 21 days after ICI initiation (11). The first schedule was recommended for part two of the trial based on safety and feasibility, but no substantial differences were observed in circulating immune cell subsets between the schedules. Such clinical study designs, integrated with mechanistic insights from optimized preclinical models, will be essential to investigate whether timing impacts therapeutic synergy and to establish rational sequencing strategies.

## Conclusion

Our study provides proof-of-concept that the ^177^Lu-labeled antibodies hG250 and rosopatamab at subtherapeutic activity levels can synergize with ICI in certain settings, supporting their potential as combination strategies to enhance efficacy while potentially reducing toxicity. However, response variability across models underscored the possible influence of tumor- and host-related immune contexts and ICI-target selection. In addition, the apparent limited impact of treatment timing should be interpreted in the context of fast-growing models. These factors should be investigated further in preclinical models incorporating mechanistic and temporal analyses. The insights gained should be integrated into the design of future clinical studies to unravel the complex interplay among TRT, ICI, and the TME. Ultimately, these efforts will be critical to guide the development of more effective and context-specific combination strategies for cancer patients.

## Data Availability

The original contributions presented in the study are included in the article/**Supplementary Material**. Further inquiries can be directed to the corresponding author.
