# Supplementary material for: Influence of immune checkpoint inhibitor target and timing on combination treatment with antibody-based β-emitting targeted radionuclide therapy across tumor immunogenicity profiles
Source: Front Immunol. 2026 Aug 4;17:1872928. doi: 10.3389/fimmu.2026.1872928 (PMC13481358; doi:10.3389/fimmu.2026.1872928)
Supplement: Supplementary file 1 [file DataSheet1.pdf]

Supplementary material

Influence of immune checkpoint inhibitor target and timing on combination treatment with antibody-based β-emitting targeted radionuclide therapy across tumor immunogenicity profiles

Simone C. Kleinendorst[1], Egbert Oosterwijk[2], Mark Konijnenberg[1,3], Milou Boswinkel[1], Manon Gloudemans[1], Sylvia T.M. Wenker[1], Stijn Muselaers[2], Peter Mulders[2], Lyndsey Maffa[4], Michael F. O'Hara[4], Michael P. Wheatcroft[4], Kwame Twumasi-Boateng[4], Sandra Heskamp[1]

[1] Department of Medical Imaging, Nuclear Medicine, Radboud University Medical Center, Nijmegen, the Netherlands.

[2] Department of Urology, Radboud University Medical Center, Nijmegen, the Netherlands.

[3] Department of Radiology and Nuclear medicine, Erasmus MC Cancer Institute, Erasmus University Medical Center, Rotterdam, The Netherlands.

[4] Telix Pharmaceuticals Limited, Melbourne, VIC 3051, Australia.

Supplementary methods

All experiments with [^{177}Lu]Lu-DOTA-hG250 were performed at Radboudumc (Nijmegen, The Netherlands) and all experiments with [^{177}Lu]Lu-rosopatamab tetraxetan ([^{177}Lu]Lu-rosopatamab) were performed at Perceptive Discovery (Needham, MA, USA).

Cell culture

Renca-hCAIX cells were cultured in RPMI1640 (Gibco) supplemented with glutamine (2 mM, Gibco), fetal calf serum (10%, FCS, Sigma-Aldrich-Chemie-BV), non-essential amino acids (NEAA, 0.1 mM Gibco), sodium pyruvate (1 mM, Gibco), and Geneticin G418 Sulphate (0.6 mg/ml, 11811-031, Gibco). MC38-hPSMA cells (Biocytogen) were cultured in DMEM with L-glutamine (Gibco) supplemented with FCS (10%, Gibco), NEAA (11140076, Gibco), HEPES (15630080, Gibco). B16F10-hPSMA cells were cultured in DMEM supplemented with 10% FCS and puromycin (1 µg/mL, A1113803, Gibco). Cells were cultured at 37°C in a humidified atmosphere with 5% CO2, and passaged maximally twelve times for all experiments.

Conjugation, radiolabeling, and quality control

hG250 was conjugated with S-2-(4-Isothiocyanatobenzyl)-1,4,7,10-tetraazacyclododecane tetraacetic acid (p-SCN-Bn-DOTA)(Macrocyclics™, Plano) and labelled with ^{177}Lu as described previously (*1,2*). In short, DOTA-conjugated hG250 was incubated with [^{177}Lu]$LuCl_3$ in 0.5 M MES buffer, pH 5.5 under metal-free conditions at 37°C for 30 minutes at 5 MBq/µg. DOTA-rosopatamab was labelled with ^{177}Lu at 0.37 MBq/µg, by incubating DOTA-rosopatamab with [^{177}Lu]$LuCl_3$ (Isotopia) in 0.1 M Ammonium Acetate pH 5.5 at 37°C for 45 minutes. Labelling efficiency of both antibodies was determined by instant thin layer chromatography (ITLC), using ITLC silica gel strip (Agilent Technologies) and 0.1 M citrate buffer (pH 6.0), and exceeded 90%. The immunoreactive fraction of [^{177}Lu]Lu-DOTA-hG250 was determined as described by Lindmo et al. (*2*) and exceeded 70%.

In vitro binding and internalization of [^{177}Lu]Lu-DOTA-rosopatamab

MC38-hPSMA and B16F10-hPSMA were seeded at 800,000 cells/well in a 6-well plate. Cells were incubated with [^{177}Lu]Lu-DOTA-rosopatamab in 1% (w/v) BSA/PBS buffer for 20, 40 or 120 minutes at 37 °C. Cells were placed on ice after incubation and washed four times in ice-cold PBS, followed by acidic glycine wash (collection of surface-bound fraction) and lysed with 0.5 M NaOH (collection of internalized fraction). Fractions were measured in a gamma counter and data was calculated as percent of total activity applied to cells. For blocking, 500 × molar excess of PSMA inhibitor 2-PMPA was used.

Animal models

Animal characteristics of the different experiments are given in **Table S1**.

MC38-hPSMA/B16F10-hPSMA

All experiments with [^{177}Lu]Lu-DOTA-rosopatamab were approved by Perceptive Discovery Institutional Animal Care and use Committee (IACUC) and executed at Perceptive Discovery (Needham, MA, USA). The experiments were performed in female C57BL/6-Folh1tm1(FOLH1)/Bcgen hPSMA transgenic mice (licensed from Biocytogen and colonies were maintained at Inotiv). Mice were accustomed to laboratory conditions for >48 hours and housed in individualized ventilated cages with ad libitum access to animal chow and water. Mice were injected with 500,000 MC38-hPSMA cells or 250,000 B16F10-hPSMA cells in 100 µL saline on the right flank under general anesthesia (isoflurane/O_2). Tumor size was measured twice a week with a digital caliper and tumor volume was

calculated with the formula $V = \frac{W^2 \times L}{2}$ (with W being width and L being length of the tumor). When MC38-hPSMA or B16F10-hPMA tumors reached a mean volume of 100-200 mm^3 or 50-100 mm^3 respectively (11 and 6-7 days post tumor injection, respectively), mice were block randomized based on tumor volume and age. If animals reached 10% loss of body weight and/or body condition score of 2.5, hydration diet gel was provided to all animals. Animals were sacrificed by CO_2/O_2 asphyxiation at the end of study or when mice reached one of the humane endpoints (tumor volume ≥ 2 cm^3 or ≥ 20 mm in any direction, tumor ulceration, ≥ 20% body weight loss compared with start weight, or severe clinical deterioration.

Renca-hCAIX

All experiments with [^{177}Lu]Lu-DOTA-hG250 were performed in accordance with the principles laid out by the revised Dutch Act on Animal Experimentation (1997), approved by the Radboudumc institutional Animal Welfare Body as described in our previous work (*1*) and executed at the Radboudumc (Nijmegen, the Netherlands). Animals were housed and fed according to Dutch animal welfare regulations. The experiments were performed in female BALB/cAnNRj, BALB/cJRj mice (Janvier, le Genest-Saint-Isle, France). Mice were accustomed to laboratory conditions for one week and housed in individualized ventilated cages with ad libitum access to animal chow and water. To reduce distress from Renca-hCAIX tumor growth, wet food was provided daily starting from tumor cell injection. Mice were injected subcutaneously with 500,000 renca-hCAIX tumor cells in 200 µL saline on the right flank. Tumor size was measured daily from 8 days post tumor injection until start of treatment and thrice a week thereafter using a digital caliper and calculating with the formula $V = \frac{4}{3}\pi abc$ (with a, b, and c being tumor radii). When Renca-hCAIX tumors reached a mean volume of 50-100 mm^3 (11-12 days post tumor injection), mice were block randomized based on tumor volume using a random number generator. Mice from different groups were housed together, cages were stored randomly, and the order in which mice were treated and measured was random. Injections and measurements were performed by a biotechnician who was blinded for group allocation. Mice without a measurable tumor or with a tumor >300 mm^3 were excluded of the experiment. On average, Renca-hCAIX tumor take was 80% . Animals were sacrificed by CO_2/O_2 asphyxiation at the end of study or when mice reached one of the humane endpoints (tumor volume ≥ 1.5 cm^3, tumor ulceration, >15% body weight loss within 2 days, >20% body weight loss compared with start weight, or severe clinical deterioration as assessed by a biotechnician blinded for group allocation). Protocols for the three therapy studies are preregistered at preclinicaltrial.eu (PCTE0000476, PCTE0000544, PCTE0000517). A priori sample size calculations were performed using an effect size of 75% decline in normalized area under the curve (nAUC) compared to control, significance level of 5% and power of 80%.

Biodistribution studies

MC38-hPSMA

Three MC38-hPSMA tumor-bearing mice were injected intravenously with 13 MBq [^{177}Lu]Lu-DOTA-rosopatamab. At day three and seven post-injection, mice were anaesthetized (isoflurane/O_2) and whole-body SPECT scans were acquired using Nanoscan (Mediso, Inc., 60-minute acquisition). Energy windows were set to 187.6-229.2 keV (primary) and 101.6-124.2 keV (secondary) and frame times were approximately 74 seconds. CT scans were acquired using a helical scan method (pitch 0.5) with 480 projections, 50 kVp tube voltage, 980 µA current, 170 ms exposure, 1:4 binning, and maximum FOV. SPECT images were reconstructed with high regularization, medium resolution (128×128), 48 iterations with a custom subset size of 3, and medium Monte Carlo quality, including attenuation and scatter correction. CT images were reconstructed using a cosine filter and voxel size of 0.25 × 0.25 × 0.25 mm^3. Mice were sacrificed on day seven and tumor and normal tissues were harvested, weighed,

and radioactivity was measured in a γ-counter (Wizard 2470; LKB/Wallace, Perkin Elmer, Boston, MA). The uptake in all organs was expressed as percentage of injected activity per gram tissue (%IA/g).

Renca-hCAIX

Protocols for biodistribution and SPECT/CT studies are preregistered at preclinicaltrials.eu (PCTE0000516, PCTE0000584). Group size was based on previous biodistribution studies (*1*). For *ex vivo* biodistribution studies, tumor-bearing mice (*n*=5/group) were injected intravenously with 0.2 MBq [^{177}Lu]Lu-DOTA-hG250 and were sacrificed at 1, 3, and 7 days post injection. Tumor and normal tissues were harvested, weighed, and radioactivity was measured in a γ-counter (1480 Wizard 3; LKB/Wallace, Perkin Elmer, Boston, MA). The uptake in all organs was expressed as percentage of injected activity per gram tissue (%IA/g). For SPECT/CT imaging, tumor-bearing mice (*n*=3) received 20 MBq [^{177}Lu]Lu-DOTA-hG250. Three days after injection, mice were anaesthetized (isoflurane/O_2) and SPECT scans were acquired using U-SPECT^{6}CT-OI (MILabs, Acquisition software v12.51, 1.6mm GPRM collimator), using the following settings: acquisition time 15 minutes per frame (4 frames), 180 seconds per bed position (6 positions). Full-body CT images were acquired in full scan angle, normal scan mode, 0.750 degrees step angle and 1x1 pixel binning, with a tube setting of 50 kV and 400 µA. Respiration of animals was continuously monitored. Scans were reconstructed with MILabs Reconstruction Software (12.00 Serial No. 80878), with a calibration factor of 4134.0 (as previously determined for this isotope/collimator combination), 0.8 mm voxel size and similarity-based ordered-subset expectation maximization algorithm at 5 iterations for SPECT images, and 80 microns for CT images. 3D maximum intensity projections were created with Imalytics Preclinical software (Gremse-IT, Aachen, Germany; Version 3.0.0.7).

Dosimetry estimations

Absorbed-dose estimations were performed using the *ex vivo* biodistribution data of renca-hCAIX tumor-bearing mice presented in figure 2D in the main article. Time-integrated activity coefficients for tumor, heart, lung, liver, spleen, pancreas, small intestine, and bone were calculated according to the trapezoid integration method (*3*). Time-integrated activity coefficients for blood, kidney, stomach, and muscle were calculated using the following formula: $TIAC[h/g] = \int_0^\infty Y_0 e^{-(K+\lambda)t} dt = \frac{Y_0}{K+\lambda}$

, with λ = decay constant [1/h], and K (= rate constant [1/h]) and Y_0 both were obtained from fitting the biodistribution data to an exponential decay equation model in Graphpad Prism. Normal organ S-values for ^{177}Lu were obtained from OLINDA/EXM 2.0 and multiplied with obtained TIACs for each organ to calculate absorbed doses according to Medical Internal Radiation Dose (MIRD) Committee methodology (*4,5*). Tumor absorbed dose was calculated by multiplying the obtained TIAC by a total conversion factor derived from the spherical model in OLINDA/EXM 2.0.

Therapy studies

Experimental details on the different animal studies performed, shown and numbered in **Figure 1**, are outlined below.

ICI-target and model-specific effects

First, we assessed the impact of ICI-target on the therapeutic outcome of combined PSMA-TRT (4 MBq [^{177}Lu]Lu-DOTA-rosopatamab) with ICI in the MC38-hPSMA model (aPD-1: study 1, CTLA-4: study 2). For the aPD-1 study, groups included (1) control, (2) TRT, (3) aPD-1 (4x), (4) TRT + aPD-1, and mice were monitored for up to 7.5 weeks. For the aCTLA-4 study, groups included (1) control, (2) TRT, (3) aCTLA-4 (2x), (4) TRT + aCTLA-4, and mice were monitored up to 6 weeks.

Second, we assessed the impact of ICI-target on the therapeutic outcome of combined CAIX-TRT (4 MBq [^{177}Lu]Lu-DOTA-hG250) with ICI in the renca-hCAIX model (study 3). Tumor-bearing mice (*n*=10/group) were randomized over the following groups: (1) control, (2) TRT, (3) aPD-1/aCTLA-4 (4) TRT + aPD-1, (5) TRT + aCTLA-4, and (6) TRT + aPD-1/aCTLA-4. Mice were monitored for up to 10 weeks.

Finally, we assessed the therapeutic efficacy of PSMA-TRT (4 or 12 MBq [^{177}Lu]Lu-DOTA-rosopatamab)) + ICI combination treatment in the B16F10-hPSMA model (study 4). Treatment groups included: (1) control, (2) 4 MBq TRT, (3) 12 MBq TRT, (4) dual-ICI, (5) 4 MBq TRT + dual-ICI, (6) 12 MBq TRT + dual-ICI. Animals were monitored up to 7 weeks.

Treatment timing effects

The fifth study assessed the therapeutic efficacy of PSMA-TRT (4 MBq [^{177}Lu]Lu-DOTA-rosopatamab) + aPD-1 combination in different treatment timing schedules. The following groups were included: (1) control, (2) TRT+ICI concurrent, (3) TRT (do), then ICI (beginning d7), (4) ICI (beginning d1), then TRT (d4), (5) ICI (beginning d1), then TRT (d5). Mice were monitored for up to 7 weeks.

The sixth study compared the therapeutic efficacy of CAIX-TRT (4 MBq [^{177}Lu]Lu-DOTA-hG250) and ICI combination therapy regimens in concurrent or TRT → ICI timing. The following groups were included: (1) control, (2) TRT+aPD-1 concurrent (3) TRT (d0), then aPD-1 (beginning d7), (4) TRT+aCTLA-4 concurrent, (5) TRT (d0), then aCTLA-4 (beginning d7). Mice were monitored for up to 6 weeks after treatment start. In this study, one mouse originally randomized to group 4 was excluded from the experiment due to an intercurrent death following the first intraperitoneal injection on day 1. To maintain a sample size of 10 in group 4, another mouse initially excluded due to a small tumor was included after its tumor had grown to 27 mm^3 by day 1. This mouse received the same injections as the others in group 4, with its treatment scheme delayed by one day.

To directly compare the therapeutic efficacy of combined CAIX-TRT with aPD-1 or aCTLA-4 with their respective mono ICI therapies, renca-hCAIX tumor-bearing mice received the same injection schedule, administration route, and doses as described for the ICI-target study in the renca-hCAIX model. The following treatment groups were included: (1) control, (2) aPD-1, (3) aCTLA-4, (4) TRT + aPD-1, or (5) TRT + aCTLA-4.

Supplementary tables

Table S1. Animal characteristics (*n* = 482)

**[^{177}Lu]Lu-DOTA-hG250 biodistribution study** (*n* = 19)				
**Characteristic**	**Day 1**	**Day 3**	**Day 7**	**Excluded animals**
BALB/cAnNRj (n)	5	5	5	4
Age at tumor injection (weeks)	13	13	13	13
Weight at tumor injection (g)	23.2 (19.6-23.3)	21.6 (20.6-23.4)	21.2 (18.2-21.8)	20.8 (19.4-21.7)
Tumor volume at day 0 (mm^3)	71 (19-162)	88 (58-153)	60 (19-115)	284 (0-641)

**[^{177}Lu]Lu-DOTA-hG250 SPECT study** (*n* = 4)		
**Characteristic**	**SPECT**	**Excluded animals**
BALB/cAnNRj (*n*)	3	1
Age at tumor injection (weeks)	14.7	14.7
Weight at tumor injection (g)	20.0 (19.8-22.2)	20.9
Tumor volume at day 0 (mm^3)	68 (88-104)	0

**[^{177}Lu]Lu-DOTA-hG250 therapy study: combination therapy regimens of TRT with various ICIs** (*n* = 66)							
**Characteristic**	**Control**	**TRT**	**aPD1-/aCTLA-4**	**TRT + aPD-1**	**TRT + aCTLA-4**	**TRT + aPD-1/aCTLA-4**	**Excluded animals**
BALB/cAnNRj (*n*)	10	10	10	10	10	10	16
Age at tumor injection (weeks)	11.4	11.4	11.4	11.4	11.4	11.4	11.4
Weight at tumor injection (g)	20 (17.7-26.4)	19.8 (17.7-24.7)	21.4 (19.2-22.5)	20.7 (18.6-23.6)	21.5 (18.5-23.3)	20.4 (18.3-22.8)	21.2 (19.4-24)

Tumor volume at day 0 (mm^3)	53 (22-184)	55 (21-190)	54 (17-190)	49 (22-234)	53 (21-229)	54 (18-195)	12 (0-368)

**[^{177}Lu]Lu-DOTA-hG250 therapy study: combined TRT+ICI treatment compared to ICI monotherapy** (*n* = 60)						
**Characteristic**	**Control**	**aPD-1**	**aCTLA-4**	**TRT + aPD-1**	**TRT + aCTLA-4**	**Excluded animals**
BALB/cJRj (*n*)	10	10	10	10	10	10
Age at tumor injection (weeks)	12	12	12	12	12	12
Weight at tumor injection (g)	24.4 (22.8-26.7)	24.1 (19-25.6)	24.8 (22.6-26.1)	24.1 (22.2-26)	24.1 (21.1-25.4)	24.7 (23-25.7)
Tumor volume at day 0(mm^3)	110 (17-236)	110 (29-251)	113 (10-222)	112 (25-221)	108 (29-223)	0.52 (0-334)

**[^{177}Lu]Lu-DOTA-hG250 therapy study: combined TRT+ICI treatment at concurrent or sequential timing** (*n* = 60)						
**Characteristic**	**Control**	**Concurrent TRT + aPD-1**	**Sequential TRT + aPD-1**	**Concurrent TRT + aCTLA-4**	**Sequential TRT + aCTLA-4**	**Excluded animals**
BALB/cAnNRj (*n*)	10	10	10	10	10	10
Age at tumor injection (weeks)	10.1	10.1	10.1	10.1	10.1	10.1
Weight at tumor injection (g)	21.1 (17.7-23)	21.1 (19.1-22)	20.5 (17.1-22)	19.9 (18.1-22)	20.3 (18.7-23)	20.8 (19.8-23)
Tumor volume at day 0 (mm^3)	61 (28-164)	63 (20-150)	62 (16-173)	51 (27-143)	60 (19-147)	0.52 (0-260)

**[^{177}Lu]Lu-DOTA-rosopatamab therapy study: combined TRT + aPD-1 in MC38-hPSMA** (*n* = 63)					
**Characteristic**	**Control**	**TRT**	**aPD-1 (4x)**	**TRT + aPD-1 (4x)**	**Excluded animals**
C57BL/6-Folh1tm1(FOLH1)/Bcgen (*n*)	10	10	10	10	23
Age at day 0 (weeks)	11 (7-18)	12 (7-21)	13 (7-19)	12 (7-21)	13 (7-21)
Weight at day 0 (g)	20 (17-22.3)	19.6 (15.4-24.9)	21 (17.1-23.8)	20.4 (16-22.3)	

Tumor volume at day 0 (mm^3)	87 (52-217)	86 (54-194)	85 (58-174)	83(57-191)	0 (0-46)

**[^{177}Lu]Lu-DOTA-rosopatamab therapy study: combined TRT + aCTLA-4 in MC38-hPSMA** (*n* = 55)					
**Characteristic**	**Control**	**TRT**	**aCTLA-4**	**TRT + aCTLA-4**	**Excluded animals**
C57BL/6-Folh1tm1(FOLH1)/Bcgen (*n*)	10	10	10	10	15
Age at day 0 (weeks)	10.6 (10.3-16)	10.9 (9.4-16)	9.4 (8.3-15.9)	10.6 (8.3-16.1)	10.6 (8.3-15.3)
Weight at day 0 (g)	18.9 (17-22.3)	20.5 (17.7-21.8)	18.3 (15.7-22.5)	19.2 (17.1-22.7)	19.4 (17.2-24.2)
Tumor volume at day 0 (mm^3)	87 (47-262)	89 (48-253)	88 (51-252)	88 (53-247)	30 (15-45)

**[^{177}Lu]Lu-DOTA-rosopatamab therapy study: combined TRT + aPD-1 in MC38-hPSMA at different timings** (*n* = 75)						
**Characteristic**	**Control**	**TRT (d0) + aPD-1 (d1-10)**	**TRT (d0) + aPD-1 (d7-17)**	**TRT (d4) + aPD-1 (d1-10)**	**TRT (d5) + aPD-1 (d1-10)**	**Excluded animals**
C57BL/6-Folh1tm1(FOLH1)/Bcgen (*n*)	10	10	10	10	10	25
Age at day -1 (weeks)	15.8 (11.4-19.4)	16.6 (11.7-19.4)	15.3 (11.7-17.4)	15.1 (11.4-19.4)	14.7 (11.4-17.4)	15.3 (11.4-19.4)
Weight at day -1 (g)	20.5 (19.2-21.5)	21.4 (19.7-25.9)	20.9 (17.2-23.9)	20.4 (17.9-24.2)	20.5 (18.1-24.6)	20.2 (17.7-23.6)
Tumor volume at day -1 (mm^3)	173 (88-240)	187 (83-283)	175 (79-256)	186 (103-266)	173 (71-287)	331.6 (71.4-579.9)

**[^{177}Lu]Lu-DOTA-rosopatamab therapy study: combined TRT + aPD-1/aCTLA-4 in B16F10-hPSMA** (*n* = 80)							
**Characteristic**	**Control**	**aPD-1/aCTLA-4**	**TRT (4 MBq)**	**TRT (12 MBq)**	**TRT (4 MBq) + aPD-1/aCTLA-4**	**TRT (12 MBq) + aPD-1/aCTLA-4**	**Excluded animals**
C57BL/6-Folh1tm1(FOLH1)/Bcgen (*n*)	10	10	10	10	10	10	20
Age at day 0 (weeks)	12.6 (12-13.4)	12.1 (11.9-15.1)	12.2 (11.9-14.3)	12.6 (12-16)	13.1 (11.9-16)	13.6 (12.1-16)	13.6 (12-16)
Weight at day 0 (g)	19.9 (18.6-23.3)	19.3 (18.1-21.3)	19.5 (17.3-21.3)	20 (18.9-22.3)	20.3 (16.2-22.1)	20.4 (18-22.9)	19.9 (18-22.8)

Tumor volume at day 0 (mm^3)	44 (25-142)	44 (26-130)	41 (19-80)	44 (19-77)	45 (28-72)	45 (29-71)	4.4 (0-23.3)

Continuous data are median and range

Table S2. *In vivo* biodistribution data of 13 MBq [^{177}Lu]Lu-DOTA-rosopatamab in male MC38-hPSMA tumor-bearing mice determined from SPECT-CT images at 3 and 7 days post injection. Data are shown as uptake (%IA/g) for 3 mice per group.

	3 days post injection Uptake (%IA/g)			7 days post injection Uptake (%IA/g)		
**Tumor**	12.2	12.5	10.8	8.7	10.9	7.5
**Heart**	6.5	8.4	7.5	3.8	4.9	4.1
**Liver**	5.3	6.8	6.0	3.8	5.2	4.3
**Spleen**	7.1	7.8	6.6	4.2	3.2	4.3
**Kidneys**	19.7	22.6	20.9	26.8	30.0	26.8
**Muscle**	1.5	1.0	1.0	1.6	0.5	0.7
**Femur**	2.2	2.0	2.3	1.6	2.1	1.8
**Deep cervical lymph nodes**	3.7	7.1	6.0	2.6	4.4	2.6
**Inguinal lymph nodes**	2.3	2.1	2.2	1.8	0.8	1.5
**Intestines**	0.8	1.2	1.2	0.8	0.9	1.1
**Lungs**	3.2	4.9	4.2	1.8	3.0	2.5
**Stomach**	1.6	2.1	0.9	1.1	1.0	1.0

Table S3. *Ex vivo* biodistribution data of 0.2 MBq [^{177}Lu]Lu-DOTA-hG250 in female renca-hCAIX tumor-bearing mice determined after dissection of organs and tumor at 1, 3, or 7 days post injection. Data are shown as uptake (%IA/g) for 5 mice per group.

	1 day post injection Uptake (%IA/g)					3 days post injection Uptake (%IA/g)					7 days post injection Uptake (%IA/g)				
**Tumor**	46.6	61.9	51.3	40.7	69.7	51.0	70.1	51.8	35.9	23.6	21.0	23.8	16.2	42.4	19.2
**Blood**	20.0	20.0	18.6	14.3	22.6	10.1	16.4	14.6	6.6	7.1	3.4	6.3	0.9	8.8	2.6
**Heart**	4.8	6.6	4.2	3.7	5.6	3.2	5.1	4.8	2.0	2.2	1.0	2.2	0.4	2.3	0.8
**Lung**	10.7	10.4	10.7	8.1	21.5	6.4	9.6	11.9	5.4	6.2	2.1	4.7	0.9	7.1	3.5
**Liver**	33.2	18.1	21.8	17.9	14.5	14.7	13.3	18.5	20.6	26.4	14.1	10.3	9.3	13.7	12.9
**Spleen**	16.1	16.2	16.6	10.8	8.8	14.6	17.1	15.4	9.6	12.6	6.8	8.2	6.1	13.3	10.0
**Pancreas**	1.7	2.4	1.7	1.4	2.4	1.3	2.0	2.3	0.9	1.1	0.5	0.8	0.3	1.1	0.6
**Kidney**	7.0	7.1	5.8	4.9	6.5	4.3	5.6	4.9	3.3	3.3	2.3	2.4	1.7	4.0	2.5
**Stomach**	2.9	2.6	2.5	1.9	3.0	1.5	2.2	1.8	1.0	1.1	0.6	0.7	0.3	1.2	0.5
**Small intestine**	2.7	6.5	4.3	3.2	5.1	2.7	3.8	3.4	2.4	2.4	1.1	1.6	0.7	2.5	1.3
**Muscle**	0.9	0.8	0.7	0.9	1.2	0.6	0.8	0.8	0.4	0.4	0.2	0.3	0.1	0.5	0.2
**Bone**	2.4	2.2	2.1	1.6	2.2	1.7	2.6	2.5	1.2	1.5	1.1	1.2	1.0	2.2	1.2

Table S4. Quantification of tumor growth and survival of therapeutic efficacy studies to the impact of ICI-target in three murine tumor models and statistical comparisons of relevant groups.

	Comparison		Tumor growth			Survival	
**Study**	**Group A**	**Group B**	**nAUC (A)**	**nAUC (B)**	**p-value**	**RMST ratio**	**p-value***
**renca-hCAIX ICI-target**	Control	TRT + aPD-1	522 (214-885)	165 (29-1007)	0.4772	3.093	0.000
		TRT + aCTLA-4		312 (163-1103)	>0.9999	1.305	0.161
		TRT + dual-ICI		50 (8-871)	0.9659	4.026	0.000
	TRT + aPD-1	TRT + dual-ICI	165 (29-1007)	50 (8-871)	0.1653	1.302	0.170
	TRT + aCTLA-4	TRT + dual-ICI	312 (163-1103)	50 (8-871)	>0.9999	3.086	0.000
	hG250 + dual-ICI	dual-ICI	171 (17-756)	284 (10-1055)	>0.9999	1.081	0.778
		TRT + dual-ICI		50 (8-871)	0.0066	0.725	0.135
**MC38-hPSMA aPD1**	Control	TRT	475 (249-663)	461 (60-968)	>0.9999	1.021	0.846
		aPD-1 (4x)		183 (11-754)	0.5197	1.091	0.416
		TRT + aPD-1 (4x)		44 (14-130)	0.0143	1.217	0.036
	TRT	TRT + aPD-1 (4x)	461 (60-968)	44 (14-130)	0.012	1.192	0.002
	aPD-1 (4x)	TRT + aPD-1 (4x)	183 (11-754)	44 (14-130)	0.8736	1.116	0.032
**MC38-hPSMA aCTLA-4**	Control	TRT	481 (37-765)	228 (10-829)	>0.9999	1.106	0.15
	Control	aCTLA-4		41 (18-98)	0.0033	1.171	0.007
	Control	TRT+aCTLA-4		33 (17-88)	0.0006	1.171	0.007
	TRT	TRT+aCTLA-4	228 (10-829)	33 (17-88)	0.0142	1.059	0.129
	aCTLA-4	TRT+aCTLA-4	41 (18-98)	33 (17-88)	>0.9999	1.059	0.129
**B16F10-hPSMA**	Control	ICI	603 (393-1165)	459 (271-724)	>0.9999	1.61	0.000
		4 MBq TRT		701 (157-920)	>0.9999	1.354	0.034
		12 MBq TRT		367 (107-727)	0.0671	1.665	0.000
		4 MBq TRT + ICI		362 (24-796)	0.1781	1.957	0.000
		12 MBq TRT + ICI		417 (235-531)	0.2408	2.165	0.000
	ICI	4 MBq TRT + ICI	459 (271-724)	362 (24-796)	>0.9999	1.216	0.116
		12 MBq TRT + ICI		417 (235-531)	>0.9999	1.345	0.001
	4 MBq TRT	4 MBq TRT + ICI	701 (157-920)	362 (24-796)	0.0454	1.446	0.022
	12 MBq TRT	12 MBq TRT + ICI	367 (107-727)	417 (235-531)	>0.9999	1.3	0.008

nAUC = normalized area under the curve, presented as median with interquartile range. P-value = adjusted for multiple comparisons by Dunn's test. RMST ratio = restricted mean survival time ratio. P-value* = tested against Bonferroni-corrected alpha values of 0.007, 0.01, 0.01, and 0.0056.

Table S5. Quantification of tumor growth and survival of therapeutic efficacy studies to the impact of treatment timing in two murine tumor models and statistical comparisons of relevant groups.

	Comparison		Tumor growth			Survival	
Study	Group A	Group B	nAUC (A)	nAUC (B)	p-value	RMST ratio	p-value*
Renca-hCAIX timing	Control	Concurrent (TRT + aPD-1)	631 (203-739)	150 (89-794)	0.0225	1.645	0.002
		Delayed (TRT + aPD-1)		278 (34-840)	0.2056	1.271	0.218
		Concurrent (TRT + aCTLA-4)		416 (15-800)	0.9765	1.318	0.191
		Delayed (TRT + aCTLA-4)		417 (4-851)	>0.9999	1.439	0.027
	Concurrent (TRT + aPD-1)	Delayed (TRT + aPD-1)	150 (89-794)	278 (34-840)	>0.9999	0.773	0.103
	Concurrent (TRT + aCTLA-4)	Delayed (TRT + aCTLA-4)	416 (15-800)	417 (4-851)	>0.9999	1.092	0.633
MC38-hPSMA timing	Control	Concurrent	692 (22-889)	221 (33-331)	0.0264	1.21	0
		Delayed		196 (18-637)	0.0796	1.21	0
		TRT offset day 4		129 (16-958)	0.0307	1.202	0
		TRT offset day 5		498 (17-734)	>0.9999	1.202	0
	Concurrent	Delayed	221 (33-331)	196 (18-637)	>0.9999	1	NaN
		TRT offset day 4		129 (16-958)	>0.9999	0.994	0.293
		TRT offset day 5		498 (17-734)	0.7029	0.994	0.293
	Delayed	TRT offset day 4	196 (18-637)	129 (16-958)	>0.9999	0.994	0.293
		TRT offset day 5		498 (17-734)	>0.9999	0.994	0.293
	TRT offset day 4	TRT offset day 5	129 (16-958)	498 (17-734)	0.7773	1	NaN

nAUC = normalized area under the curve, presented as median with interquartile range. P-value = adjusted for multiple comparisons by Dunn's test. RMST ratio = restricted mean survival time ratio. P-value* = tested against Bonferroni-corrected alpha values of 0.0083 and 0.0056.

Supplementary figures

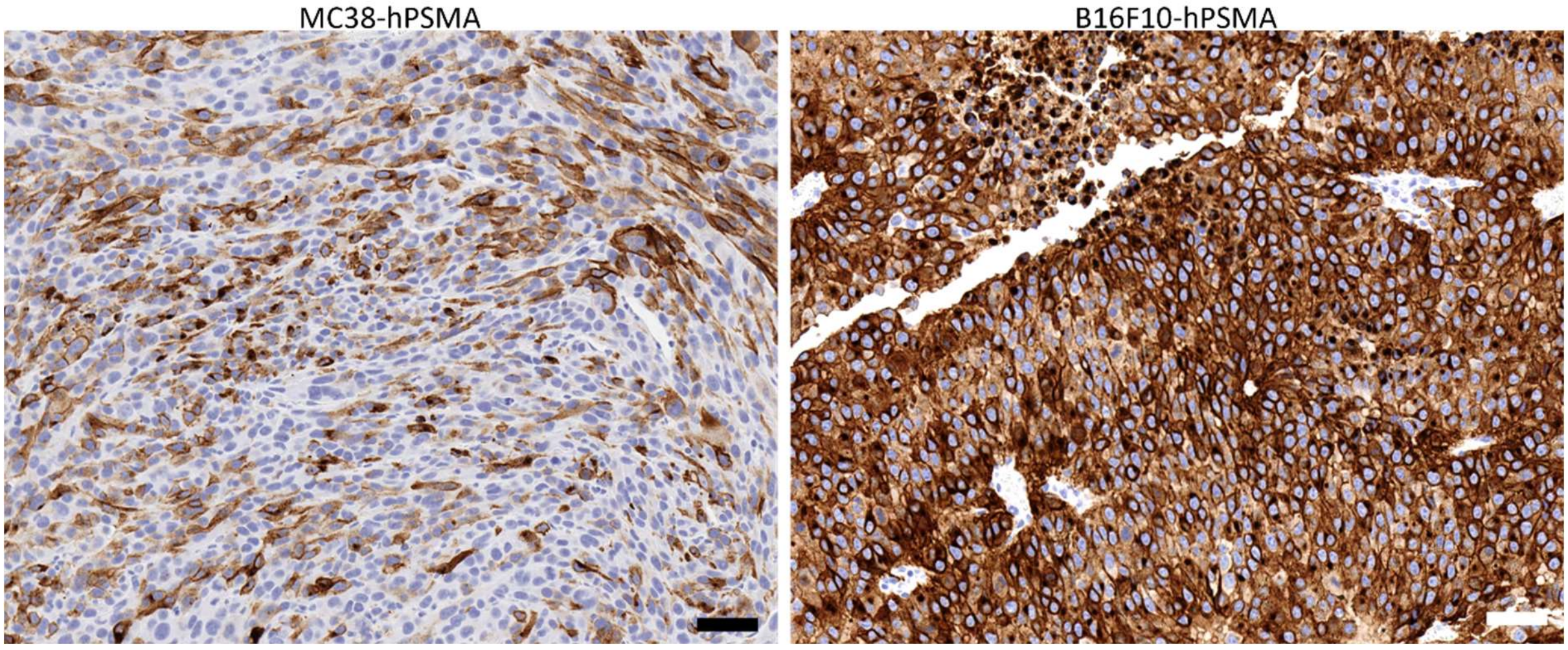

Figure S1. Immunohistochemical PSMA staining confirms PSMA expression in MC38-hPSMA (left) and B16F10-hPSMA (right) tumors. Images were captured at 20x resolution.

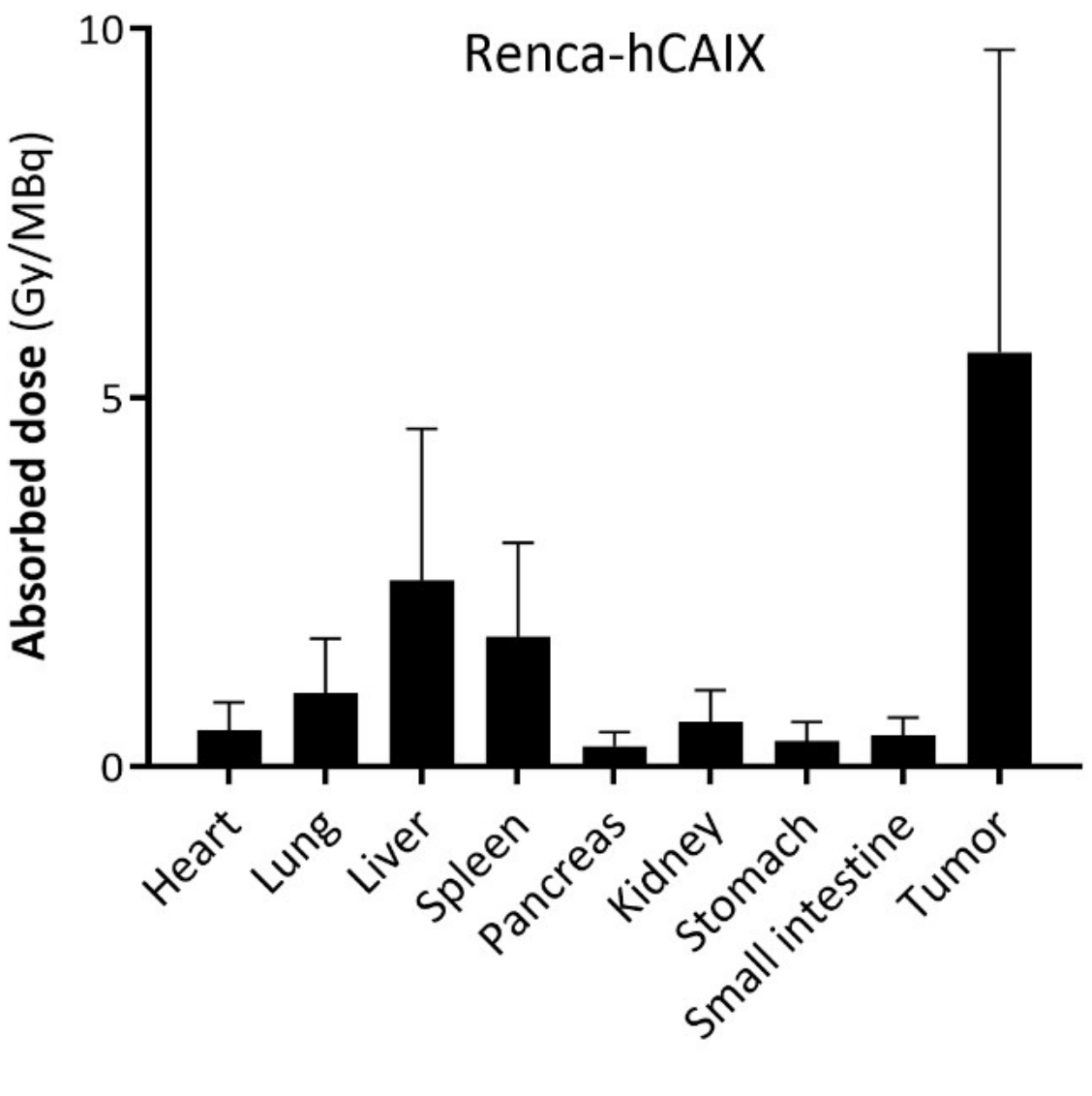

Figure S2. Absorbed dose to organs and renca-hCAIX tumor of [^{177}Lu]Lu-DOTA-hG250.

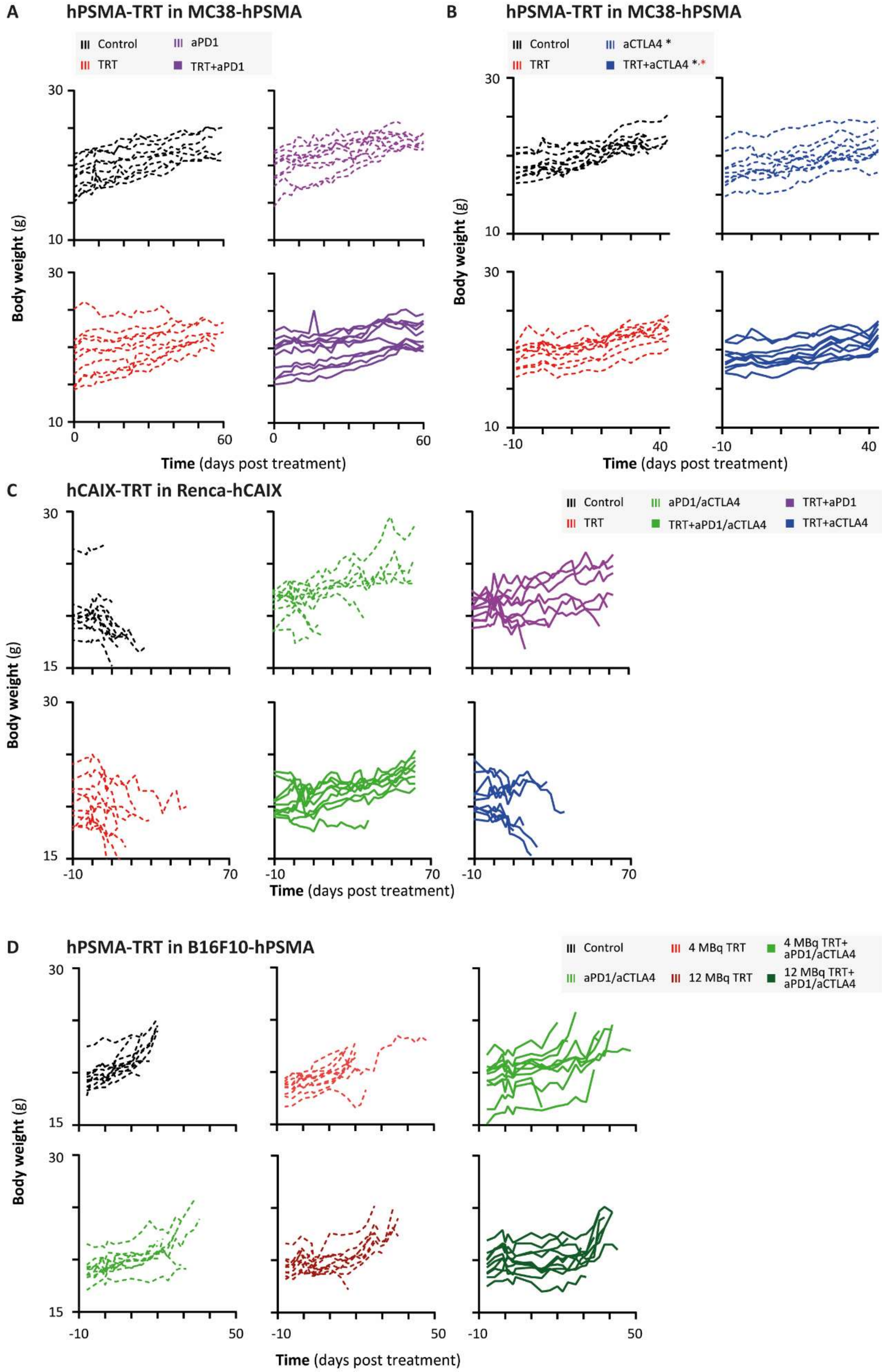

Figure S3. Body weights of (**A-B**) MC38-hPSMA tumor-bearing mice treated with 4 MBq [177Lu]Lu-DOTA-rosopatamab + aPD-1 (4x) or aCTLA-4 (2x), (**C**) renca-hCAIX tumor-bearing mice treated with 4 MBq [177Lu]Lu-DOTA-hG250 + aPD-1, aCTLA-

4 or dual-ICI (8x), and (**D**) B16F10-hPSMA tumor-bearing mice treated with 4 or 12 MBq [^{177}Lu]Lu-DOTA-rosopatamab + dual-ICI (4x).

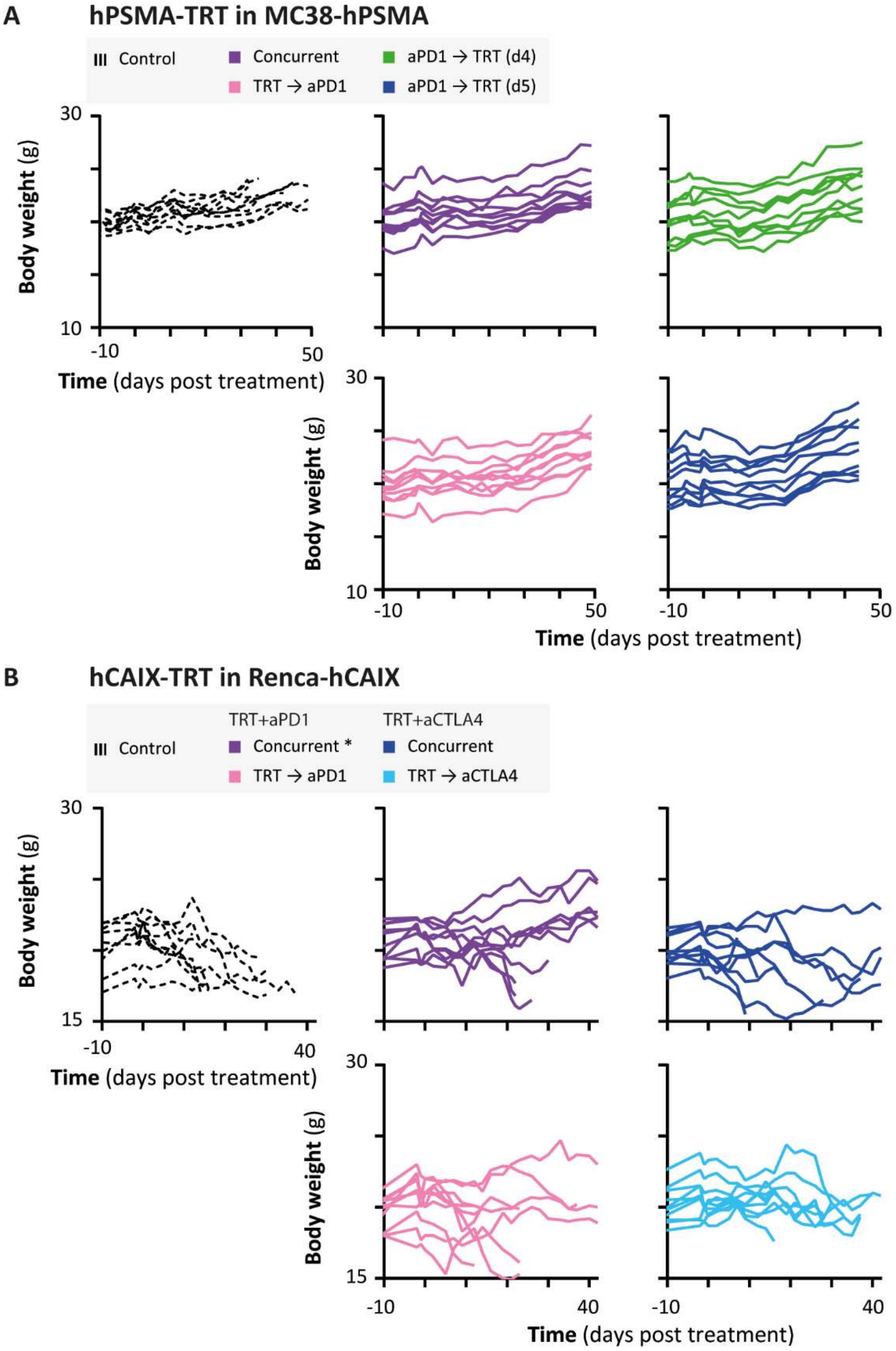

Figure S4. Body weights of (**A**) MC38-hPSMA tumor-bearing mice treated with 4 MBq [^{177}Lu]Lu-DOTA-rosopatamab + aPD-1 (4x) at different time schedules, and (**B**) renca-hCAIX tumor-bearing mice treated with 4 MBq [^{177}Lu]Lu-DOTA-hG250 + aPD-1 or aCTLA-4 (8x) at different time schedules.

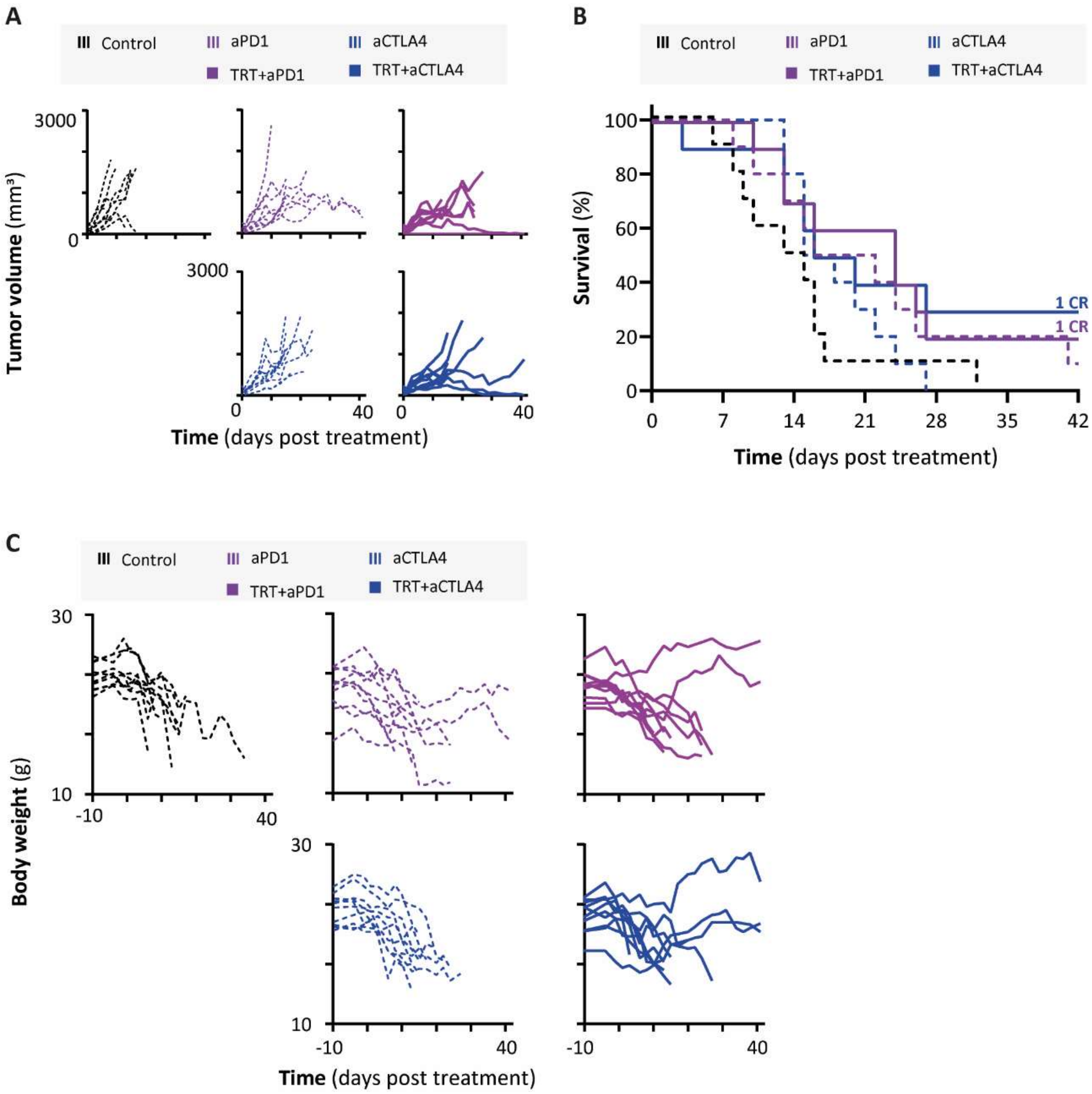

Figure S5. Therapeutic efficacy of 4 MBq [^{177}Lu]Lu-DOTA-hG250 with aPD-1 or aCTLA-4 (8x) compared with ICI monotherapies in renca-hCAIX tumor-bearing mice, evaluated by (**A**) tumor growth and (**B**) overall survival, and (**C**) overall wellbeing of the mice monitored by body weight.

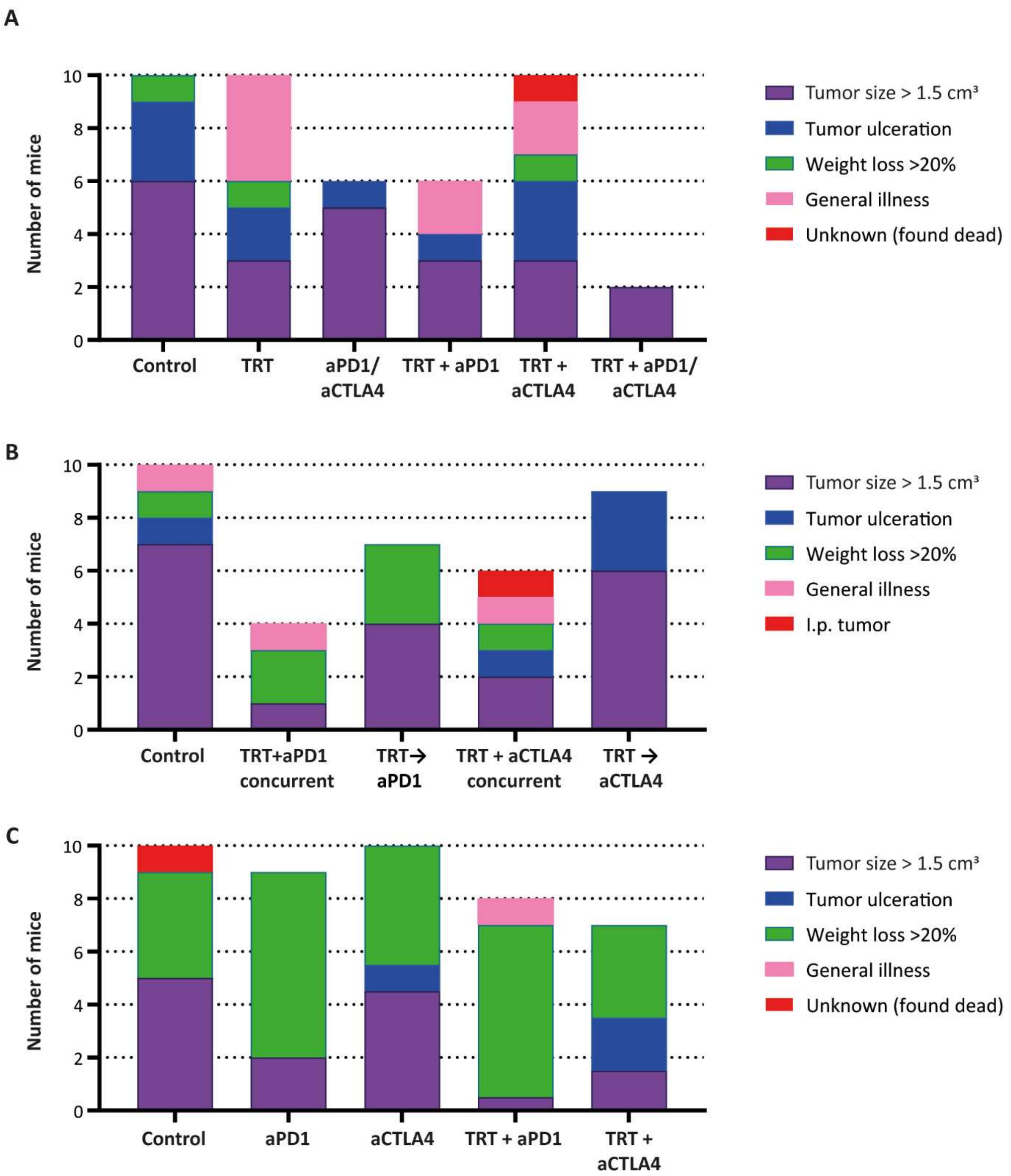

Figure S6. Overview of the humane endpoints that were applied with the therapy studies on renca-hCAIX tumor bearing mice (**A-C**), showing a higher frequency of weight loss in the experiment directly comparing combination therapy to aPD-1 or aCTLA-4 monotherapy (**C**).

References

1. Kleinendorst SC, Oosterwijk E, Molkenboer-Kuenen J, et al. Towards effective CAIX-targeted radionuclide and checkpoint inhibition combination therapy for advanced clear cell renal cell carcinoma. *Theranostics.* 2024;14:3693-3707.

2. Lindmo T, Boven E, Cuttitta F, Fedorko J, Bunn PA, Jr. Determination of the immunoreactive fraction of radiolabeled monoclonal antibodies by linear extrapolation to binding at infinite antigen excess. *J Immunol Methods.* 1984;72:77-89.

3. Konijnenberg M. From imaging to dosimetry and biological effects. *Q J Nucl Med Mol Imaging.* 2011;55:44-56.

4. Bolch WE, Eckerman KF, Sgouros G, Thomas SR. MIRD pamphlet No. 21: a generalized schema for radiopharmaceutical dosimetry--standardization of nomenclature. *J Nucl Med.* 2009;50:477-484.

5. Stabin MG, Xu XG, Emmons MA, Segars WP, Shi C, Fernald MJ. RADAR reference adult, pediatric, and pregnant female phantom series for internal and external dosimetry. *J Nucl Med.* 2012;53:1807-1813.
